# Editorial: Clinical implementation of genetic scores in cardiovascular medicine

**DOI:** 10.3389/fcvm.2023.1292116

**Published:** 2023-11-24

**Authors:** Maya S. Safarova

**Affiliations:** Division of Cardiovascular Medicine, Department of Medicine, Froedtert and Medical College of Wisconsin, Milwaukee, WI, United States

**Keywords:** precision medicine, prevention, GWAS - genome-wide association study, Mendelian randomization, monogenic (Mendelian) traits, polygenic risk assessment, genetic risk score (GRS), atherosclerosis

**Editorial on the Research Topic** Clinical implementation of genetic scores in cardiovascular medicine

## Introduction

**Figure 1:**
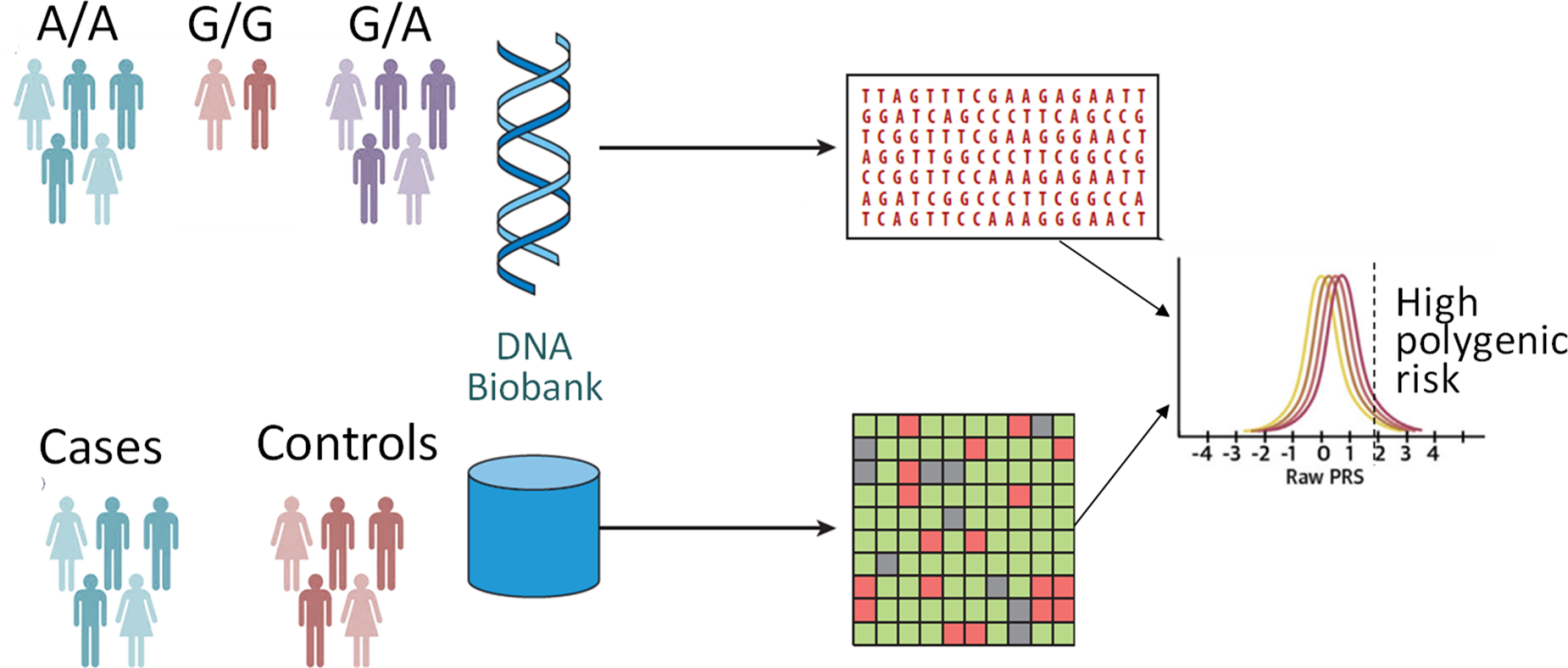
Polygenic risk analyses. A polygenic score represents a single value estimate of an individual's genetic liability to a trait or disease. It is calculated by the sum of an individual's risk alleles, weighted by risk allele effect sizes derived from genome-wide association study data.

The paradigms for precision cardiovascular medicine are undergoing continuous evolution and growth. Recent technological and computational discoveries allowed the first look into a complete, gapless genome sequence (1), offering new insights into the epigenetics of aging. With ongoing investigations of the role of genetic risk integrated into clinical assessment, current recommendations offer biomarker- and imaging-based risk-enhancing factors to individualize the approach to each patient. Most of the cardiovascular traits have polygenic underpinning which can be implicated in the heterogeneity of prognosis of those with monogenic disorders. Genome-wide association studies (GWAS) identified millions of single-nucleotide polymorphisms allowing genetic risk modeling of a given disease state or phenotype. Polygenic risk scores (PRSs) can be calculated as a weighted sum of the number of trait-associated alleles for each individual (Figure 1). PRS is a powerful tool which aggregates the many small effects of variants across the genome to estimate an individual's disease risk as a single quantitative risk factor (2). A PRS is typically normally distributed and those at the tails of the distribution may have a significantly greater or lower risk of disease than those in the middle. There is limited data on the validation and implementation of PRS in clinical practice and populations with ancestral and ethnic diversity (3). In this issue of the *Journal*, studies focusing on the genetic basis and architecture of cardiovascular diseases and novel applications of precision medicine tools were reviewed.

## Application of genetic scores in unrelated traits

Polygenic scores (PGSs) have transformed genetic research studies allowing exploration of shared genetic components between distinct disorders. As such, McElligott et al. in a longitudinal population-based prospective study of 483,177 participants of the UK Biobank demonstrated clinically significant difference in the rates of cardiovascular complications in patient with sepsis, using phenotype-specific PRSs. In individuals of the European ancestry, these individual PRSs for myocardial infarction, stroke, and venous thromboembolism may provide individualized management guidance, especially within the first 30 days of sepsis diagnosis. Yun et al. applied genome-wide PRSs in the UK Biobank cohort of middle aged individuals of the European ancestry to demonstrate an independent 2.6-fold and 2.1-fold increase in the likelihood of cardiovascular mortality among those with high genetic risk for coronary heart disease and type 2 diabetes, respectively. The authors also provided validation of prior findings in individualizing risk optimization through behavioral and therapeutic interventions to reduce cardiovascular mortality risk (4, 5). The investigators approached the heterogeneity of cardiometabolic risk showing the importance of genetic and lifestyle factors in susceptibility to cardiovascular mortality.

## Implementation of statistical genetics relevant to cardiovascular phenotypes

Bidirectional mendelian randomization approach uses genetic variants as an instrument to determine whether an association between a risk factor/marker and an outcome is causal in nature and whether the marker causes the outcome, or the outcome influences the marker (6). This method assumes single temporal direction between the risk factor and the outcome. Using telomeres as the marker of biological aging, Sha et al. provided insights into association between leukocyte telomere length and atrial fibrillation in participants of the FinnGen and UK Biobanks. The authors strengthened their analytics with the sensitivity analysis, revealing causal effect of genetically predicted atrial fibrillation on the shortening of the telomere length. There was no causal effect of genetically predicted telomere length on the risk of atrial fibrillation. These findings demonstrate potential clinical value in early detection and treatment of atrial fibrillation to prevent leukocyte telomerase length shortening. Using a cross-lagged panel model and focusing on the elderly women of the East Asian descent, Wu et al. showed clinical significance of the *LPL* gene in the risk assessment of metabolic syndrome in individuals with non-alcoholic fatty liver diseases.

## Review of methods papers and tools to facilitate individualized care

Beyond risk prediction, genetic evaluation provides insights into disease prognosis, mechanisms and subtypes, causality through Mendelian randomization studies and pleiotropy through phenome-wide association studies. Gianazza et al. discuss clinical application of the targeted proteomics for discovery and validation of candidate protein biomarkers. There is continuous interest in agnostic evaluation of clinically relevant therapeutic targets. While a phenome-wide association study approach leverages electronic health record data (7), the authors review the role of proteomics and lipidomics in discovering pleiotropic effects of proprotein convertase subtilisin/kexin type 9 (PCSK9), one of the key regulators of the low-density lipoprotein receptor metabolism. Further, the investigators discuss the role of combined multiomics approach with whole-genome sequencing data in atherosclerosis and related traits. PRSs may help explain variability in penetrance and expressivity of monogenic disorders. Chumakova and Baulina provide insights into the missing heritability problem in patients with hypertrophic cardiomyopathy. The authors review scenarios of recessive and *X*-linked patterns of inheritance for this in general autosomal-dominant condition as well as impact of polygenic contribution to the disease development.

## Considerations for clinical practice

Public health strategies effectively providing early risk stratification and prevention have the potential to advance cardiovascular health worldwide. The predictive value of PGS incrementally increases with the use of multiple scores in combination with clinical and environmental factors. As PGSs continue to improve performance, we will see widespread and cost-effective implementation of PGSs for monogenic as well as polygenic cardiovascular conditions. There is a need for the development of artificial intelligence algorithms to accommodate methods that integrate PRSs and other genetic risk information, family history, and clinical variables. Such comprehensive risk assessment with a linkage to electronic health record-based strategies is expected to increase awareness, detection, and control of various cardiovascular conditions with greater accuracy. PRSs can be leveraged to predict variation in drug response and susceptibility to adverse drug reactions. Therefore, research focusing on the use of PRSs to inform targeted therapies on the basis of cardiovascular disease pathways is needed. The cost-effectiveness of such strategies along with the use of PRSs to enrich clinical trials with those at higher polygenic risk or variable drug efficacy is needed. Health history collected in electronic health records and health registries, by wearables and other automated lifestyle trackers along with integrating the full allele frequency spectrum into the PRS algorithms can be utilized for developing comprehensive targeted risk communication tools. There is a crucial need in developing and validating PRSs generated from various ancestry groups using novel statistical genetics approaches aimed at the reduction rather than the promotion of health disparities. Ethical, legal, and social issues remain relevant in the polygenic context in the practice of cardiovascular medicine and public health.

In conclusion, building public trust and engagement, providing structured training for healthcare providers (8), and bringing together specialists in population genetics, translational genomics, implementation science, ethics, cost and outcomes researchers is needed for PGSs to be adopted in clinical practice.
